# Examining adherence barriers among women with HIV to tailor outreach for long-acting injectable antiretroviral therapy

**DOI:** 10.1186/s12905-020-01011-8

**Published:** 2020-07-25

**Authors:** Lorie Benning, Andrea Mantsios, Deanna Kerrigan, Jenell S. Coleman, Elizabeth Golub, Oni Blackstock, Deborah Konkle-Parker, Morgan Philbin, Anandi Sheth, Adaora A. Adimora, Mardge H. Cohen, Dominika Seidman, Joel Milam, Seble G. Kassaye, Tonya Taylor, Miranda Murray

**Affiliations:** 1 Department of Epidemiology, Johns Hopkins Bloomberg School of Public Health, Baltimore, MD USA; 2 Independent Consultant, New York, NY USA; 3 Center for Health, Risk and Society, American University, Washington, DC USA; 4 Department of Gynecology and Obstetrics, Johns Hopkins School of Medicine, Baltimore, MD USA; 5 Montefiore Medical Center, Albert Einstein College of Medicine, New York, NY USA; 6 Division of Infectious Diseases, University of Mississippi Medical Center, Jackson, MS USA; 7 Columbia University Mailman School of Public Health, Sociomedical Sciences, New York, USA; 8 Department of Medicine, Division of Infectious Diseases, Emory University School of Medicine, Atlanta, Georgia; 9 Department of Medicine, School of Medicine and Department of Epidemiology, UNC Gillings School of Global Public Health, University of North Carolina at Chapel Hill, Chapel Hill, NC USA; 10 Department of Medicine, Stroger Hospital, Cook County Bureau of Health Services, Chicago, IL USA; 11 Department of Obstetrics, Gynecology & Reproductive Sciences, University of California, San Francisco, California USA; 12 Institute for Health Promotion and Disease Prevention Research, University of Southern California, Los Angeles, CA USA; 13 Division of Infectious Diseases and Travel Medicine, Georgetown University, Washington, DC USA; 14 SUNY Downstate Medical Center, Brooklyn, NY USA; 15 Independent Consultant, London, UK

**Keywords:** HIV, ART, Long-acting injectable, Adherence, Women

## Abstract

**Background:**

Long-acting (LA) injectable antiretroviral therapy (ART) has been found non-inferior to daily oral ART in Phase 3 trials. LA ART may address key barriers to oral ART adherence and be preferable to daily pills for some people living with HIV. To date, women have been less represented than men in LA ART research. Using longitudinal data from the Women’s Interagency HIV Study (WIHS) cohort of women living with HIV in the United States, we examined barriers and facilitators of daily oral ART adherence that may be related to or addressed by LA ART.

**Methods:**

We conducted a secondary analysis of WIHS cohort data from 1998 to 2017 among participants seen for at least 4 visits since 1998 who reported using ART at least once (*n* = 2601). Two dichotomous outcomes, patient-reported daily oral ART adherence and viral suppression were fit using generalized linear models, examining the role of socio-demographic and structural factors.

**Results:**

At study enrollment, the median age was 40.5 years, 63% of participants were African American and 22% were Latina. The majority (82%) reported taking ART more than 75% of the time and 53% were virally suppressed. In multivariate analysis, several sub-groups of women had lower odds of reported adherence and viral suppression: 1) younger women (adherence aOR: 0.71; viral suppression aOR: 0.63); 2) women who inject drugs (adherence aOR: 0.38; viral suppression aOR: 0.50) and those with moderate (adherence aOR: 0.59; viral suppression aOR: 0.74) and heavy alcohol consumption (adherence aOR: 0.51; viral suppression aOR: 0.69); 3) those with depressive symptoms (adherence aOR: 0.61; viral suppression aOR: 0.76); and 4) those with a history of going on and off ART (adherence aOR: 0.62, viral suppression aOR: 0.38) or changing regimens (adherence aOR: 0.83, viral suppression aOR: 0.56).

**Conclusions:**

Current injectable contraceptive users (vs. non-users) had greater odds of oral ART adherence (aOR: 1.87) and viral suppression (aOR: 1.28). Findings identify profiles of women who may benefit from and be interested in LA ART. Further research is warranted focused on the uptake and utility of LA ART for such key subpopulations of women at high need for innovative approaches to achieve sustained viral suppression.

## Background

The effective use of anti-retroviral therapy (ART) among people living with HIV (PLHIV) has dramatically reduced AIDS-related morbidity and mortality [1–3], while simultaneously reducing sexual transmission of the virus to others [4, 5]. Despite the promise of increased access to and use of ART across settings and populations over time, both HIV treatment and prevention outcomes remain suboptimal due in part to barriers related to consistent adherence to daily oral ART [6–9]. Switching from multiple tablets, often several times a day, to a single tablet regimen has been found to improve adherence and virologic suppression, however optimal adherence remains a problem for many people currently on daily oral ART [10]. Lack of ART adherence can also lead to viral resistance, making HIV infection more difficult to treat.

Research suggests that 40% of PLHIV in the United States (U.S.) who are in care have some degree of ART non-adherence [11, 12]. A variety of factors are significantly associated with sub-optimal adherence, including: demographics (gender, age), clinical factors (e.g. side effects, pill burden), psychosocial factors (e.g. not taking drugs when one doesn’t feel sick, depression/anxiety, and perceived stigma and discrimination), and structural factors (e.g. food security, transportation costs) [13–17]. Research suggests that adherence continues to be a major issue particularly for women living with HIV [18, 19]. Studies in the U.S. and internationally have documented lower ART adherence in women than men [20–22]. The gender differences observed in ART adherence are often attributed to inequitable gender norms and the roles and responsibilities that women have inside and outside the home [21, 23]. Race and ethnicity are also associated with lower ART adherence among Black and Latino PLHIV [24–27]. Black and Latina women are affected by racism and related structural factors as well as gender norms, contributing to complex and multi-level barriers to ART adherence for women in these sub-groups [28–30].

A new method of delivery, long-acting (LA) injectable ART, offers hope for addressing some of the aforementioned oral ART adherence issues and is currently being evaluated in Phase III clinical trials [31]. LA ART would require monthly or every 2 month injections, eliminating the need for daily pills. By providing a potentially more convenient and private option for accessing ART and being preferable to daily pills for some PLHIV, LA ART may improve individual and population-level HIV outcomes. Several ongoing studies are evaluating LA ART using two drugs - Cabotegravir, a DNA integrase inhibitor, and Rilpivirine, a reverse transcriptase inhibitor. To date, LA ART has been proven non-inferior to daily oral ART (e.g. equivalent levels of viral suppression) in completed Phase II and ongoing Phase III trials [31, 32]. The majority of LA ART trial participants have thus far been male. Given that LA ART may soon become an option in routine care, it is critical to better understand its possible role among women living with HIV considering both preferences and needs of diverse subpopulations. We conducted a secondary analysis of data from the Women’s Interagency HIV Study (WIHS) to examine barriers and facilitators to ART adherence in women, with attention to those that may be particularly well addressed by LA ART.

## Methods

### Study design

The WIHS is an observational study and the largest ongoing prospective cohort study of HIV among women in the U.S. Our analytic sample contained ten WIHS consortia located in Bronx/Manhattan, NY; Brooklyn, NY; Los Angeles/Southern California/Hawaii; San Francisco/Bay Area, CA; Chicago, IL; Washington, DC; Atlanta, GA; Chapel Hill, NC; Miami, FL; and Birmingham, AL/ Jackson, MS. The WIHS study design and cohort profile have been described in detail in previous publications [33–35]. There have been four enrollment waves since the WIHS began in 1993: 1.) 1994–1995; 2.) 2001–2002; 3.) 2011–2012; and 4.) 2013–2015. WIHS semi-annual study visits include clinical exams, blood collection, and interviewer-administered questionnaires to collect information about sociodemographics, substance use, HIV medication use including adherence. This analysis included women with HIV who participated for a minimum of four semi-annual study visits between October 1998 and March 2017 and who reported using ART at least once (*n* = 2601). Therefore, inclusion in the current analysis included participants who were followed for a minimum of one and a half years (wave 4: 2013–2015) to a maximum of 18 years (wave 1 from 1998).

### Primary outcome measures

The two primary outcomes were self-reported ART adherence and viral suppression. Self-reported ART adherence was determined at each semi-annual visit by participant response to the question, “*In general, over the past six months, how often did you take your antiretrovirals as prescribed*?” Possible response options included 100% of the time [1], 95–99% of the time [2], 75–94% of the time [3], < 75% of the time [4], I haven’t taken any of my prescribed medications [5]. Responses were re-coded and dichotomized with 1–3 counted as adherent and 4–5 counted as non-adherent. This categorization was used as current ART regimens, especially those with Integrase Strand Transfer Inhibitors (INSTI), require approximately 75% adherence to achieve 90% viral suppression [36–38].

HIV-1 RNA viral load was quantified for all HIV-infected WIHS participants at each semi-annual study visit. For visits prior to October 1, 2008, WIHS utilized the NucliSens assay (Organon Teknika Corporation [OTC], Durham, NC; Nowicki 2001) with a lower limit of quantification (LLQ) of 80 copies/ml. Beginning October 1, 2008, WIHS utilized the COBAS AmpliPrep/COBAS Taqman HIV-1 Test (Roche Molecular Systems, Branchburg, NJ) with LLQ = 48 copies/ml through March 31, 2011 and LLQ = 20 copies/ml beginning April 1, 2011. Given the clinical goal of ART is to achieve viral suppression below a given assay’s limit of detection, viral loads were dichotomized using the highest limit of 80 copies/ml and those below that limit were counted as being virally suppressed.

### Independent variables and measures

Independent variables included five key domains: sociodemographic and study characteristics, ART regimen and adherence experiences, prior injection experience, mental health, and substance abuse. Sociodemographic characteristics included: age, race, education, marital status, housing (stable vs. unstable) and employment (employed vs. unemployed), annual household income (dichotomized at $24,000 cut-point), health insurance (insured vs. uninsured), and WIHS enrollment wave.

ART regimen and adherence measures included length of time on ART, regimen type by class (e.g. protease inhibitors (PI), non-nucleoside reverse transcriptase inhibitors (NNRTI), entry inhibitors (EI), integrase inhibitors (II), number of regimen switches, and type of regimen change (re-start from being off ART at previous visit or different regimen from previous visit). Experiences using injections included prior and current injection drug use, prior and current use of injectable contraception (depo medroxyprogesterone acetate) and prior and current use of injectable insulin. The mental health measure included in analysis was reported depressive symptoms using the Center for Epidemiologic Studies Depression Scale (CES-D) [39] and the substance use measures included reported cigarette, alcohol and illicit drug use.

### Statistical analyses

Standard descriptive methods were used to analyze baseline data. Continuous variables were summarized using the number of observations, mean, median, standard deviation and interquartile range. Categorical variables were summarized using the number of observations and percentages. Both dichotomous primary outcomes were fit using generalized linear models, specified with the binomial distribution and a logit link and with generalized estimating equations used to adjust standard errors to account for repeated measures [40]. Thirty datasets were generated using single-chain Markov-chain Monte Carlo multiple imputation methods to complete missing data on covariates separately for each visit. Models were run for each of the 30 imputed data sets and results were combined using Rubin’s estimator of the variance [41]. Analyses were conducted in SAS, Version 9.4. *P*-values < 0.05 were considered to be statistically significant.

### Ethical considerations

WIHS participants provided written informed consent and were compensated for their participation in the study. The WIHS protocol has been approved by the Institutional Review Board at each study site’s institution and by the WIHS executive committee. Data are collected at clinical sites and entered into a password-secured web-based data entry system maintained by MACS/WIHS Combined Cohort Study Data Analysis Coordinating Center staff at Johns Hopkins University. Raw data from questionnaires, clinical exam forms and laboratory result forms are run through two rounds of edits and then summarized semi-annually. More detailed information is available at https://statepi.jhsph.edu/wihs/wordpress/.

Data used in the current analysis were de-identified. This study was considered to be exempt by the Institutional Review Board of the Johns Hopkins Bloomberg School of Public Health. This secondary analysis used previously collected, anonymized data. No identifying information was accessed.

## Results

### Socio-demographic characteristics

At baseline, the median age among the subset of the cohort included in this analysis was 40.5 years (Table 1). Almost two-thirds (63%) of the women were African American and 22% were Latina. Approximately one third reported having less than high school education. Among the sample, 5% had unstable housing, two-thirds (67%) were unemployed and 79% had an annual income less than or equal to $24,000. A total of 9% of women did not have health insurance while the remaining 91% had government-funded health care programs such as Medicaid, Medicare and the Ryan White HIV/AIDS Program, and private insurance.

**Table 1:** Baseline sociodemographic and biobehavioral characteristics of WIHS participants

Factor	***N*** = 2601
**Socio-demographic and study characteristics**	
Median age (IQR)	40.5 (34.5, 47.1)
Race/ethnicity	
African American, non-Hispanic	1632 (63)
Latina/Hispanic	574 (22)
White, non-Hispanic	317 (12)
Asian/Pacific Islander/Native American or Alaskan/Other	78 (3)
Enrollment wave	
Wave 1: 1994–1995	1230 (47)
Wave 2: 2001–2002	617 (24)
Wave 3: 2011–2012	230 (8)
Wave 4: 2013–2015	524 (20)
Less than high school education	949 (36)
Married or partnered	863 (33)
Unstable housing	140 (5)
Unemployed	1754 (67)
Annual household income ≤$24,000	2057 (79)
No health insurance	241 (9)
Pregnant in past 6 months	114 (4)
**Mental Health**	
Depressive symptoms (CES-D score ≥ 16)	1043 (40)
**Substance Use**	
Current smoker	1196 (46)
Alcohol use	
None	1401 (54)
Low (> 0–7 drinks per week)	965 (37)
Moderate (> 7–12 drinks per week)	97 (4)
Heavy (> 12 drinks per week)	138 (5)
Non-injection drug use	617 (24)
Injection drug use	
Never	2018 (78)
Former	528 (20)
Current	55 (2)
**Medical injection experience**	
Insulin use (medical injection)	
Never	2525 (97)
Former	15 (1)
Current	61 (2)
Depo medroxyprogesterone acetate use (medical injection)	
Never	2300 (88)
Former	156 (6)
Current	145 (6)
**Adherence and viral suppression**	
≥ 75% adherence reported	2135 (82)
HIV RNA ≤80 copies/ml	1370 (53)

### Mental health, behavioral and ART adherence factors

Depressive symptoms, as indicated by CES-D ≥ 16, were reported by 40% of participants. At baseline, 46% were current smokers, 46% reported any alcohol use in the past 6 months, 24% reported non-injection illicit drug use in the past 6 months, 20% reported previous injection drug use and 2% were currently injecting drugs. In terms of experience with medical injections, 2% were currently using insulin injections and 6% were currently receiving depo medroxyprogesterone acetate injections. There was high reported adherence to daily oral ART but low levels of viral suppression at baseline: 82% reported taking ART more than 75% of the time but only 53% were virally suppressed.

**Fig. 1:**
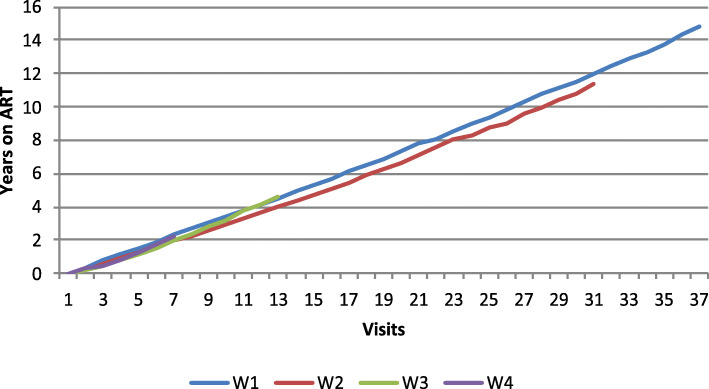
Years on ART by wave

**Fig. 2:**
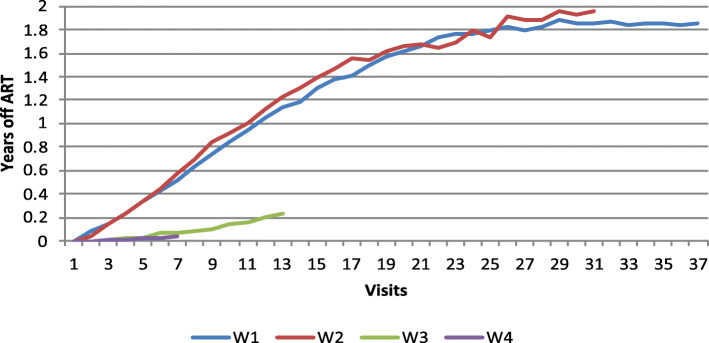
Years off ART by wave

**Fig. 3:**
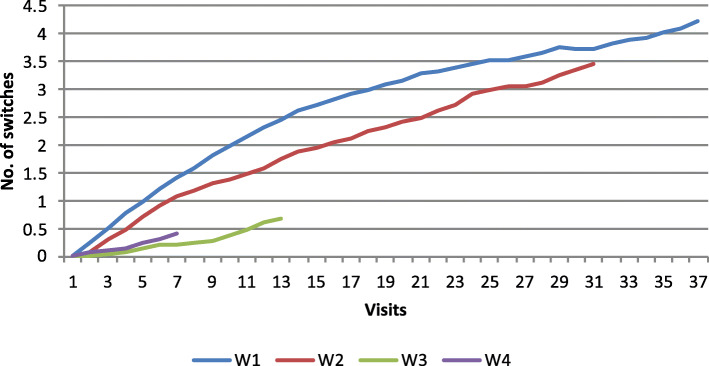
Number of ART switches by wave

Figures 1, 2 and 3 show adherence to ART and treatment switches, based on wave of enrollment. As shown in Fig. 1, time on ART is consistent across enrollment waves, except for women enrolled in 2001–2002 (Wave 2), who had lower average years on ART than women enrolled in the other waves. As seen in Fig. 2, women who enrolled earlier, in 1994–1995 (Wave 1) and 2001–2002 (Wave 2) had significantly more treatment discontinuations from ART with averages of up to 2 years off of ART while women enrolled in 2001–2012 (Wave 3) and 2013–2015 (Wave 4) had far less time off of ART, indicating a shift over time to improved treatment adherence. Figure 3 shows that there were distinct patterns of ART switching by wave, with overall fewer numbers of switches among women enrolled in waves 3 and 4 than in women in earlier waves.

### Factors associated with adherence and viral suppression

In multivariate analysis, several socio-demographic characteristics were associated with adherence to ART and viral suppression (see Table 2). Later enrollees (2001 onwards) were more likely to be suppressed compared to 1994–1995 enrollees. Better adherence and viral suppression were associated with older age (adherence aOR: 1.41; viral suppression aOR: 1.59 per 10 years) and being married/partnered (adherence aOR: 1.28; viral suppression aOR: 1.18). Reported adherence and viral suppression were lower among African American women (adherence aOR: 0.62; viral suppression aOR: 0.71) and Latina/Hispanic women (adherence a OR: 0.76; viral suppression aOR: 0.84) compared to White women. Women who reported substance use were less adherent and less likely to be virally suppressed than those who reported no use. Women who currently smoked had lower odds of being adherent (aOR: 0.77) and suppressed (aOR: 0.67). Moderate drinkers had a lower odds of being adherent (aOR: 0.59) and lower odds of being virally suppressed (aOR: 0.74). Similarly, heavy drinkers were less adherent (aOR: 0.51) and less virally suppressed (aOR: 0.69). Women who reported illicit drug use but did not inject also had lower odds of adherence (aOR: 0.68) and viral suppression (aOR: 0.93) than women with no reported use, as did women who reported currently injecting drugs (adherence aOR: 0.38; viral suppression aOR: 0.50).

**Table 2:** Multivariate model of factors associated with adherence and viral suppression

Factor	Adherence		Viral suppression	
	aOR	95% CI	aOR	95% CI
**Demographic and study characteristics**				
Number of abbreviated visits (per visit)	**0.95**	**0.91–0.98**	**1.10**	**1.07–1.13**
Number of missed visits (per visit)	**0.97**	**0.95–1.00**	0.99	0.97–1.01
Age (per 10 yr.)	**1.41**	**1.32–1.49**	**1.59**	**1.54–1.64**
Race/ethnicity (vs. White, non-Hispanic)				
African American, non-Hispanic	**0.62**	**0.53–0.72**	**0.71**	**0.66–0.77**
Latina/Hispanic	**0.76**	**0.64–0.90**	**0.84**	**0.77–0.91**
Asian/Pacific Islander/Native American or Alaskan/Other	1.10	0.79–1.52	0.99	0.85–1.15
Enrollment wave (vs. 1994–1995 northern site recruits)				
2001–2002 northern site recruits	1.07	0.97–1.20	**1.75**	**1.65–1.85**
2011–2012 northern site recruits	**1.85**	**1.44–2.39**	**3.29**	**2.89–3.75**
2013–2015 southern site recruits	**1.86**	**1.47–2.36**	**4.61**	**4.05–5.25**
Less than high school education	0.95	0.87–1.05	1.04	0.99–1.09
Married or partnered	**1.28**	**1.16–1.42**	**1.18**	**1.12–1.24**
Unstable housing	0.87	0.71–1.06	0.96	0.85–1.09
Unemployed	0.95	0.85–1.06	**0.78**	**0.72–0.82**
Annual household income ≤$24,000	**1.14**	**1.00–1.29**	**0.86**	**0.81–0.92**
No health insurance	0.85	0.69–1.04	0.91	0.81–1.03
Pregnant in past 6 months	1.15	0.85–1.55	0.93	0.80–1.09
Depressive symptoms (CES-D score ≥ 16)	**0.61**	**0.56–0.67**	**0.76**	**0.73–0.80**
**Substance use**				
Current smoker	**0.77**	**0.70–0.85**	**0.67**	**0.63–0.70**
Alcohol use (vs. None)				
Low (> 0–7 drinks per week)	**0.88**	**0.79–0.97**	0.95	0.90–1.01
Moderate (> 7–12 drinks per week)	**0.59**	**0.47–0.73**	**0.74**	**0.65–0.85**
Heavy (> 12 drinks per week)	**0.51**	**0.43–0.60**	**0.69**	**0.62–0.78**
Non-injected illicit drug use	**0.68**	**0.61–0.76**	**0.93**	**0.87–0.99**
Injected illicit drug use (vs. Never)				
Former	0.94	0.84–1.06	**0.91**	**0.86–0.97**
Current	**0.38**	**0.30–0.49**	**0.50**	**0.41–0.61**
**Medical injection experiences**				
Insulin use (medical injection; vs. Never)				
Former	1.00	0.77–1.29	**1.15**	**1.00–1.31**
Current	1.01	0.76–1.35	*1.15*	*0.99–1.33*
Depo medroxyprogesterone acetate use (medical injection; vs. Never)				
Former	0.92	0.81–1.05	1.05	0.98–1.12
Current	**1.87**	**1.39–2.53**	**1.28**	**1.12–1.46**
**ART adherence characteristics**				
Cumulative time on ART > cumulative time off ART	**1.32**	**1.12–1.56**	**0.82**	**0.74–0.91**
Type of regimen switch (vs. same regimen as previous visit)				
Re-start (off ART at previous visit)	**0.62**	**0.52–0.74**	**0.38**	**0.34–0.43**
Switch (different regimen than previous visit)	**0.83**	**0.72–0.95**	**0.56**	**0.52–0.60**

Depressive symptoms were associated with lower adherence (aOR: 0.61) and viral suppression (aOR: 0.76). Women with a history of “treatment holidays” were less adherent (0.62) and less virally suppressed (0.38) than women who did not stop treatment, as were women with a history of changing ART regimens (adherence aOR: 0.83; viral suppression aOR: 0.56). Women who used depo medroxyprogesterone acetate (injectable contraceptive) had a greater odds of daily oral ART adherence (aOR: 1.87) and viral suppression (aOR: 1.28) compared to women who did not.

## Discussion

This study examined barriers to daily oral ART adherence among 2601 women living with HIV in the WIHS cohort who reported using ART at least once since 1998, with the goal of assessing opportunities for LA ART. Cohort members were comprised of women from across the 10 WIHS consortium clinical subsites, representing the population of women living with HIV in each of the 10 metropolitan areas across the U.S. This sample was largely comprised of African American and Latina women with lower socio-economic status. In general, we found that the odds of adherence to daily oral ART increased from 2001 onwards. This coincides with the initiation of highly active antiretroviral therapy (HAART) and subsequent changes to ARV treatments, specifically, a shift over time in guidelines around when to start and if to stop treatment as it became clear that episodic antiretroviral therapy was significantly less effective than continuous ART [42]. Study findings indicate that lower adherence to daily oral ART and lower odds of viral suppression were associated with younger age, substance use, depressive symptoms, and ART regimen changes. Use of injectable contraceptives was associated with greater odds of adherence and viral suppression. These findings have important implications, as LA ART may address adherence barriers and meet patient needs and preferences among women who have difficulty being adherent to an oral regimen or who have experience with injectable contraception.

Younger women living with HIV may benefit most from LA ART. Underscoring the findings in this study, previous research indicates that younger age is associated with suboptimal adherence [43–45]. Factors such as stigma and social pressure [46, 47], depression [46, 48], and competing daily demands [46, 49, 50] have all been found to be associated with lower adherence among youth. Prior research also indicates that youth who are newly initiating treatment and going through medication changes [51] and those who have higher number of medications prescribed [49] and complicated/burdensome treatment regimens [50, 52] may also be less adherent. LA ART could address several of these identified barriers to treatment adherence that youth face. Offering less frequent treatment with monthly or bi-monthly injections rather than daily pills and a less complicated regimen – receiving a healthcare-provider administered injection rather than having to remember to take one or multiple pills daily – could facilitate better adherence among youth. In qualitative research conducted with LA ART clinical trial participants exploring appropriate patient populations for this treatment modality, participants identified youth as particularly well-suited for LA ART given that younger patients are less accustomed to taking pills and have difficulty adhering to oral regimens [53, 54].

Based on findings that people who use substances are less adherent to ART [55–58], this is another subgroup of women who could also be well served by LA ART. Among people living with HIV who use drugs, higher adherence to oral ART has been found in those who receive care in structured settings, such a directly observed therapy [59, 60], suggesting the healthcare provider-administered injections of LA ART may be a good fit for this population. On the other hand, receiving an injection may be a triggering event for some of these individuals and careful consideration should be given in order to avoid potential relapse.

Consistent with the current findings, both depression and depressive symptoms are risk factors for ART non-adherence [55, 56, 61, 62] presenting another target group for whom LA ART may be a good option. When asked about candidates for the injectable option, LA ART clinical trial participants identified individuals with mental health conditions as those who may benefit from this option citing that people suffering from depression related to their overall health, HIV status, or self-identity as a patient, could be liberated from the daily reminder of pill-taking [53, 54].

Study findings also indicate that women experiencing changes in their ART regimen (going on and off regimens and switching regimen type) may benefit from LA ART. Treatment disruptions may occur for various reasons including treatment fatigue, side effects and lifestyle changes. Given low rates of adverse events and high rates of patient satisfaction among Phase II clinical trial participants [32], LA ART may present a regimen option that is more sustainable for some women living with HIV, ensuring that they are more likely to remain on it without disruption and thus improve their overall adherence and treatment outcomes.

A particularly salient study finding is that women receiving periodic injections for contraceptive use (depo medroxyprogesterone acetate) were more likely to be adherent to oral ART. For this sub-group, the convenience of and familiarity with periodic injections may make LA ART appealing given their experience with injectable contraceptives. Given the higher levels of ART adherence detected in this analysis among this subgroup of women, they may choose to continue with oral ART or consider injectable ART where periodic injections and appointments are required. In prior qualitative research with LA ART and PrEP clinical trial participants, the use of depo medroxyprogesterone acetate as an ongoing form of injectable contraception among women was compared by both female and male participants and study investigators to the potential use of a periodic injectable ART regimen [63, 54].

PLHIV in the LA ART clinical trials noted that feeling supported by and comfortable with their providers played a role in adherence to their monthly clinic appointments for injections [54]. The importance of a good patient-provider relationship for individuals returning to the clinic has implications for HIV-related health outcomes for PLHIV. If LA injection appointments provide an opportunity for more provider involvement in the lives of PLHIV who feel supported by having regular interactions with the healthcare community, this treatment modality could not only address adherence barriers by improving likelihood of participants returning to clinic for injections but also help providers identify and address other health problems and concerns among women living with HIV through more frequent patient interactions.

Our study findings identify profiles of women with suboptimal adherence and viral suppression who may be particularly interested in and benefit from expanded options for HIV treatment, including LA ART. These findings raise important questions around the implementation of this treatment modality in real-world settings outside of clinical trials given the subsets of women identified here as potential candidates. While younger women, those with a history of injection experiences as well as those who suffer from depression, may benefit from or be interested in an injectable ART option, a real-world challenge will be how to ensure that they return to the clinic regularly for injection appointments.

This study has limitations. We relied on self-reported adherence and included a period in the early 2000s when potential benefits of switching and intermittent discontinuation were being investigated in the Strategies for Management of Antiretroviral Therapy (SMART) Study [42]. It is possible, but unknown, whether some WIHS participants were participants in this study or that their clinical care was based on its rationale. In this respect, discontinuation may have been prescribed and thus might not have been non-adherence, as we have counted it. Additionally, we were unable to adjust for dosage, pill burden, and other reasons for discontinuation or regimen switch. The length of the study period and the contribution of information from multiple enrollment waves has both limitations and strengths in that our analysis is impacted and reflects shifts in treatment options and the evolution of advances in prescribing practices of ART. Furthermore, the diversity of demographic, behavioral and clinical data available point to profiles of women who likely would not meet the selection criteria for clinical trials like the SMART Study [64].

Treatment success can be optimized by providing expanded options for ART. Certain sub-sets of women adhere well to an oral regimen while others may face challenges. With more choices, women will be able to find treatment options that best fit their needs, abilities, preferences, and situations and thus facilitate adherence and viral supression.

## Conclusions

Opportunities for LA ART to address adherence barriers and patient needs and preferences exist among women who may have difficulty being adherent to an oral regimen or who have experience receiving injectable contraception. This analysis provides insights into the diverse subsets of women living with HIV who may benefit from and appreciate the choice of LA ART. Further research is needed to understand how women, transitioning from oral to LA ART can best be supported to adhere to injection appointments, to ensure optimal treatment outcomes. This is especially relevant to an important segment of the population of women living with HIV who are from lower socio-economic backgrounds and may benefit from additional services to ensure optimal ART adherence.

## Data Availability

The datasets used and/or analysed during the current study are available from the MACS/WIHS Combined Cohort Study with approval from the Executive Committee.

## Abbreviations

AIDS: Acquired immunodeficiency syndrome
aOR: Adjusted odds ratio
ART: Antiretroviral therapy
ARV: Antiretroviral
CES-D: Center for Epidemiologic Studies Depression Scale
CI: Confidence interval
DNA: Deoxyribonucleic acid
EI: Entry inhibitors
HAART: Highly active antiretroviral therapy
HIV: Human immunodeficiency virus
II: Integrase inhibitors
INSTI: Integrase strand transfer inhibitors
IQR: Interquartile range
LA: Long-acting
NNRTI: Non-nucleoside reverse transcriptase inhibitors
PI: Protease inhibitors
PLHIV: People living with HIV
RNA: Ribonucleic acid
SMART: Strategies for Management of Antiretroviral Therapy Study
WIHS: Women’s Interagency HIV Study
